# Case Report: Convalescent Plasma Achieves SARS-CoV-2 Viral Clearance in a Patient With Persistently High Viral Replication Over 8 Weeks Due to Severe Combined Immunodeficiency (SCID) and Graft Failure

**DOI:** 10.3389/fimmu.2021.645989

**Published:** 2021-05-03

**Authors:** Verena Keitel, Johannes Georg Bode, Torsten Feldt, Andreas Walker, Lisa Müller, Anselm Kunstein, Caroline Klindt, Alexander Killer, Tina Senff, Jörg Timm, Philipp Ostermann, Maximilian Damagnez, Nadine Lübke, Ortwin Adams, Heiner Schaal, Gerald Antoch, Jennifer Neubert, Philipp Albrecht, Sven Meuth, Saskia Elben, Annemarie Mohring, Johannes C. Fischer, Edwin Bölke, Manfred Hoenig, Ansgar S. Schulz, Tom Luedde, Björn Jensen

**Affiliations:** ^1^Department of Gastroenterology, Hepatology and Infectious Diseases, University Hospital Duesseldorf, Medical Faculty, Heinrich-Heine-University Duesseldorf, Duesseldorf, Germany; ^2^Institute of Virology, University Hospital Duesseldorf, Medical Faculty, Heinrich-Heine-University Duesseldorf, Duesseldorf, Germany; ^3^Department of Diagnostic and Interventional Radiology, University Hospital Duesseldorf, Medical Faculty, Heinrich-Heine-University Duesseldorf, Duesseldorf, Germany; ^4^Department of Pediatric Oncology, Hematology and Clinical Immunology, University Children’s Hospital, Medical Faculty, Heinrich-Heine-University Duesseldorf, Duesseldorf, Germany; ^5^Department of Neurology, University Hospital Duesseldorf, Medical Faculty, Heinrich-Heine-University Duesseldorf, Duesseldorf, Germany; ^6^Department of Hematology, Oncology and Clinical Immunology, Medical Faculty, Heinrich-Heine-University Duesseldorf, Duesseldorf, Germany; ^7^Institute for Transplant Diagnostics and Cell Therapeutics, University Hospital Duesseldorf, Medical Faculty, Heinrich-Heine-University, Duesseldorf, Germany; ^8^Department of Radiation Oncology, University Hospital Duesseldorf, Medical Faculty Heinrich-Heine-University, Duesseldorf, Germany; ^9^Pediatric Stem Cell Transplantation Unit, University Hospital Ulm, Ulm, Germany

**Keywords:** SARS-CoV-2, severe combined immunodeficiency, humoral immune response, convalescent plasma, remdesivir

## Abstract

We describe the unique disease course and cure of SARS-CoV-2 infection in a patient with SCID and graft failure. In absence of a humoral immune response, viral clearance was only achieved after transfusion of convalescent plasma. This observation underscores the necessity of the humoral immune response for SARS-CoV-2 clearance.

## Introduction

We describe a 25-year-old female patient with severe combined immunodeficiency (SCID) due to a RAG1 variant (1, 2) with persistently high SARS-CoV-2-RNA concentrations in respiratory samples over 60 days. Immunocompromised patients have not only an increased risk of acquiring severe Corona virus disease 2019 (COVID-19) (3, 4) but may fail to achieve viral clearance with prolonged shedding of viable virus (5, 6).

Our patient was first treated with remdesivir and subsequently received convalescent plasma (CP), which achieved sustained viral clearance.

## Case Description and Diagnostic Assessment

The patient was diagnosed with T^-^/B^-^/NK^+^ SCID and received unconditioned haploidentical hematopoietic stem cell transplantation (HSCT) from her father at 4 months of age (7). Due to incomplete immune reconstitution with poor T cell- and no B cell-engraftment she received a stem cell boost without preconditioning at 4 years of age, repetitive donor lymphocyte infusions (5 times, last infusion 11/2019) and regular immunoglobulin substitution therapy.

She suffered from recurrent bronchopulmonary infections and chronic obstructive pulmonary disease. Due to progressive graft failure she was scheduled for another HSCT.

After a close friend tested positive for SARS-CoV-2, testing was performed while she was asymptomatic and results were positive for SARS-CoV-2 on 30^th^ of April 2020 (day 0). Since patients with SCID are prone to severe systemic viral infections (e.g. cytomegalovirus, adenovirus, parainfluenza virus) (8–10) she was admitted for clinical observation.

**Figure 1:**
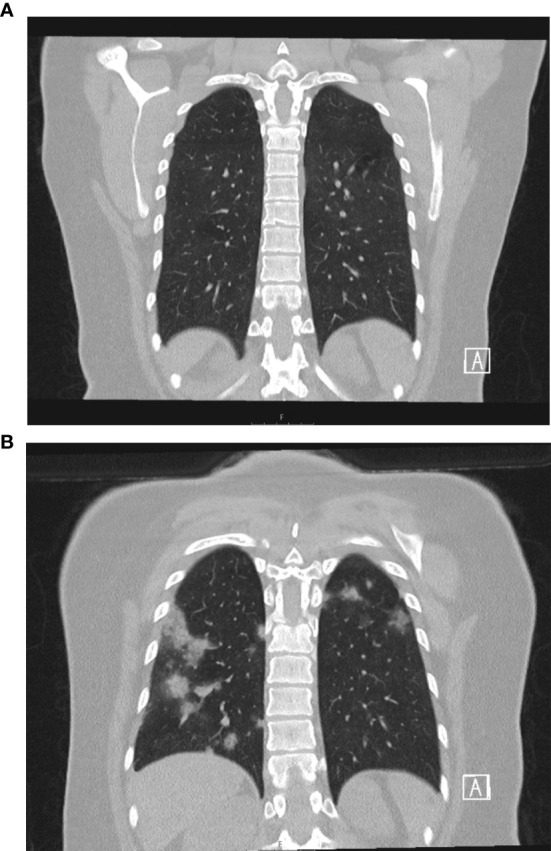
Chest CT scans on day 3 after admission **(A)** without signs of COVID-19 and day 34 **(B)** showing COVID-19 pneumonia.

Upon admission, her physical examination, vital signs, chest radiography and a CT scan were unremarkable (**Figure 1**). The patient experienced a mild headache for one day but no other COVID-19 associated symptoms. The initial SARS-CoV-2-RNA concentration in the nasopharyngeal swab was 4.89 x 10^8^ copies/ml. SARS-CoV-2 could not be PCR-amplified from the patient’s EDTA blood, bone marrow, urine and stool samples. Over the course of 30 days, the patient did not develop any overt symptoms despite persistent high-level viral replication.

On initial admission (day 0) the patient had a reduced neutrophil count (nadir of 115/µl on day 4), lymphopenia (389/µl) with reduced T-cells 250/µl (CD4^+^CD45RA^+^T-cells 6.4/µl; CD4^+^CD45RO^+^T-cells 63/µl; CD8^+^CD45RA^+^T-cells 29/µl; CD8^+^CD45RO^+^T-cells 68/µl). NK-cells (CD3^-^CD56^+^) were reduced to 1.3% (4.8/µl). Monocytes were 285/µl and B-cells were absent, which was in line with undetectable IgA and IgM levels (IgG was substituted). Neutrophils were reduced shortly after infection and recovered preceding development of pneumonia (**Table 1**). The patient received prophylactic antibiotic and antifungal treatment.

**Table 1:** Laboratory and virological findings; n.d., not detected; NPS, nasopharyngeal swab; CRP, C-reactive protein; PCT, procalcitonin; WBC, white blood cell count (absolute numbers) and differentiation by FACS.

	01/2019	d1	d4	d14	d21	d33	d43	d46	d54/55	d64	d75	d82	d109
Viral load NPS *10^6^	not appl.	490	116	227	202	19	0.5	0.1	148	n.d.	n.d.	n.d.	n.d.
CRP (mg/dl) <0.5		0.8	0.6	3.4	0.3	4.4	0.3	0.2	0.3	<0.1	<0.1	0.1	0.5
PCT (ng/ml) <0.05		0.07	0.07	0.03	0.03	0.07		0.1	0.08	0.06	0.05	0.04	
IL-6 (pg/ml)		3.9				24.6	9.5		5.3				
Ferritin (µg/ml)		33	77	69	29	90	87	47	32	42	26	24	19
WBC *10^4^/µl	5.5	0.8	0.6	1.0	2.6	3.5	3.1	4.9	3.5	4.9	3.4	4.0	4.5
Neutrophils (n/µl)		190	125	1238	1134	2479	1135	1928	1322	2628	1623	2329	3045
CD20+ B-cells (n/µl)	n.d.	n.d.	n.d.	n.d.	n.d.	n.d.	n.d.	n.d.	n.d.	n.d.	n.d.	n.d.	n.d.
CD3+ T-cells (n/µl)	574	250	373	428	522	375	435	617	816	676	711	709	1151
CD3+/CD4+ (n/µl)	125		71	72	92	56	88	96	114	97	92	118	152
CD3+/CD8+ (n/µl)	224		108	86	187	157	215	327	426	358	343	339	530
CD3-/CD56+/ CD16+ (n/µl)	79		4.8	7.3	7.3	5.2	6.1	16.2	19.3	16.7	6.21	12.0	21.5

Yellow indicates values before SARS-CoV-2 infection. Grey indicates remdesivir application (d33-d43), green indicates application of 6 units of convalescent plasma (CP) from 2 different donors (d55-d64).

**Figure 2:**
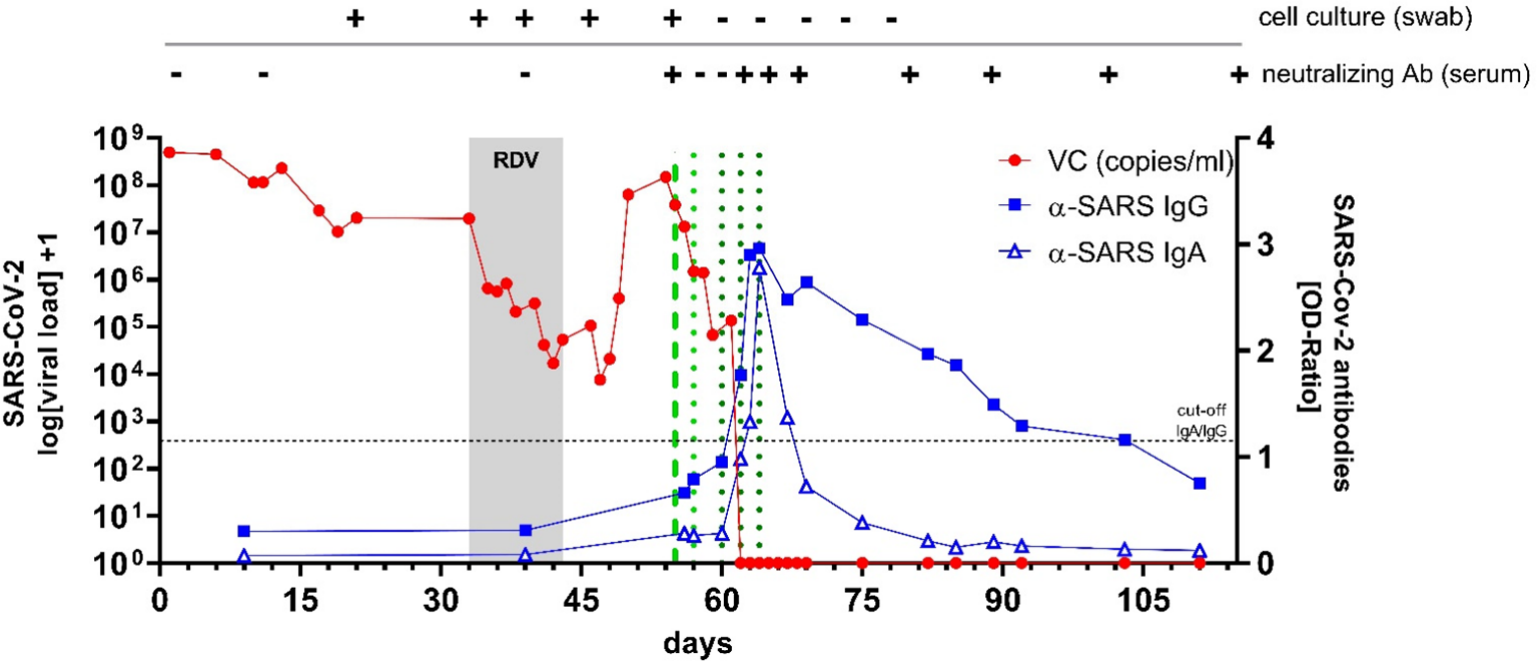
SARS-CoV-2 viral concentration (VC) in nasopharyngeal swabs (in red) and spike specific SARS-CoV-2 antibody titers over time (in blue). Cell culture was used to determine viral replication as well as presence of neutralizing antibodies (Ab). Application of remdesivir (RDV) over 10 days is depicted in grey. Application of convalescent plasma from 2 different donors is depicted as dotted green lines (light green = 3 units from donor-1; dark green = 3 units from donor-2).

On d33 of follow-up the patient presented without overt symptoms, but oxygen saturation was 93% and a CT-scan showed signs of COVID-19 pneumonia (**Figure 1**). SARS-CoV-2-RNA was 1.95 x 10^7^ and 4.07 × 10^6^ copies/ml in nasopharyngeal and bronchial fluid samples, respectively. Thus, COVID-19 pneumonia was diagnosed and the patient received remdesivir (200 mg i.v. on d33, 100 mg/d i.v. d34-42) over 10 days (11). Remdesivir treatment reduced viral concentrations from 1.95 x 10^7^ copies/ml to 5.35 x 10^4^ copies/ml (**Figure 2**). Whole genome sequencing of SARS-CoV-2 showed no remdesivir resistance development. Clinical symptoms of pneumonia improved, however, virus concentrations increased again to levels of 1.48 x 10^8^ copies/ml on d54. To achieve viral clearance, the patient received two units of convalescent plasma (CP, 250 ml each) from donor-1 on day 55 (12). This contained spike-specific IgA- and IgG-antibodies (OD-ratios were 1.94 and 3.26, respectively) and had a neutralizing antibody titer (NT-titer) of 1:80. On d57 a third unit of donor-1 CP was administered. Viral concentration dropped from 3.8 x 10^7^ copies/ml (d55) to 6.75 x 10^4^ copies/ml (d59, 2.75-log reduction). Infusion of three additional units of CP from a different donor (donor-2; d60, d62, d64; IgA/IgG OD-ratio: 8.58/6.44; NT-titer: 1:80) resulted in undetectable viral concentration on NP swabs and increased anti-SARS-CoV-2 antibodies in the patient’s serum above the detection limit (IgA/IgG OD-ratio: 2.78/2.96) (**Figure 1**). The patient’s symptoms cleared completely and SARS-CoV-2 RNA remained negative even after anti-SARS-CoV-2 antibodies decreased below the detection limit on day 111. The patient received the planned second HSCT on day 138 following conditioning with treosulfan (42g/m^2^). Despite this immunosuppressive and -modulatory procedure, the SARS-CoV-2-RNA was not detected by PCR on NP swabs or in the patient’s blood (last test from day 158).

## Discussion

This unique case illustrates the course of COVID-19 in a situation where the functionality of innate and especially adaptive humoral and cellular immunity is severely limited. Development of COVID-19 pneumonia was significantly delayed despite high viral concentrations and only developed after partial recovery of the cellular immune response. As expected, viral clearance is not achieved with severely impaired T-cell and absent B-cell mediated responses (13, 14). This case and the detection of viral replication in cell culture beyond d50 highlights the need for prolonged quarantine measures and monitoring in patients with immune defects (6).

While remdesivir treatment reduced virus concentrations by 2.6-log, however, after stopping of the drug virus concentrations quickly recovered. CP administration from two different donors achieved sustained viral clearance even after anti-SARS-CoV-2 antibodies dropped below the detection limit, which is in line with reports from patients with primary and secondary immunodeficiency as well as with hematological malignancies (15–17). This therapeutic effect was retained even during a second HSCT on day 138. This case report underscores the importance of the humoral immune response, substituted here by CP transfusions, to successfully clear SARS-CoV-2 infection.

## Data Availability Statement

The datasets presented in this study can be found in online repositories. Consensus Sequences are available on GISAID: EPI_ISL_572330, EPI_ISL_572331, EPI_ISL_572333, EPI_ISL_573152, EPI_ISL_574259, EPI_ISL_572397. See also **Supplementary Material**.

## Ethics Statement

The examinations were carried out in accordance with the Declaration of Helsinki and the patient gave written informed consent for use of CP as well as for publication of the pseudonymized results and patient history.

## Author Contributions

VK, JB, TF, and BJ initiated this work, supervised the study, and drafted the manuscript. VK, JB, TF, BJ, AKu, CK, AKi, TL, AM, AS, MH, PA, GA, JN, SM, and SE took care of the patient, analyzed the clinical data and phenotype, determined diagnostic procedures and treatment plan, and interpreted treatment responses. AW, LM, TS, JT, PO, MD, NL, OA, and HS developed virological test strategies (ELISAs, testing for neutralizing SARS-CoV-2 antibodies, viral sequencing), and performed and interpreted virological data. All authors critically revised the manuscript. All authors contributed to the article and approved the submitted version.

## Funding

JT received acknowledges funding through BMBF B-FAST.

## Conflict of Interest

The authors declare that the research was conducted in the absence of any commercial or financial relationships that could be construed as a potential conflict of interest.
